# Detection and reconstruction of tandemly organized de novo copy number variations

**DOI:** 10.1186/1471-2105-11-S11-S12

**Published:** 2010-12-14

**Authors:** Dan He, Nicholas Furlotte, Eleazar Eskin

**Affiliations:** 1 Dept. of Comp. Sci., Univ. of California Los Angeles, Los Angeles, CA 90095, USA

## Abstract

**Background:**

The characterization of structural variations (SV) such as insertions, deletions and copy number variations is a critical step in the process of understanding the full genetic architecture of organisms. Copy number variations (CNV) have attracted much recent attention due to their effects on gene expression and disease status.

**Results:**

In this paper, we present a method that utilizes next-generation sequencing technologies (NGS), in order to both detect and reconstruct CNVs. We focus on a special type of CNV, namely tandemly organized *de novo* CNVs, which have been shown to occur with high frequency in the mouse genome.

**Conclusions:**

We apply our method to CNV regions randomly inserted into the reference mouse genome and show that our method achieves good performance for both detection and reconstruction of tandemly organized *de novo* CNVs.

## Background

Structural variations (SVs), such as insertions, deletions, and copy number variations (CNVs), have been shown to account for a large portion of genetic variance in both mouse and human genomes [1][2]. SVs are also known to contribute to phenotypic variation and have been implicated in a number of diseases [3]. Therefore these genetic variants can be utilized in a fashion similar to that of SNPs and may be useful when conducting association studies aimed at explaining mechanisms of complex diseases [4]. CNVs have been shown to make up around 12% of the human genome and various studies show their presence can affect gene expression, cause disease and alter the organism’s phenotype [5][6][7][8]. For this reason, the problem of CNV detection has attracted a lot of recent attention. The efforts of many recent studies have been aimed at the detection of SVs and the prediction of their genomic regions [9][1][10][11][12][13][2]. However, the general problems of detecting and especially reconstructing CNVs still lack effective methods.

There have been several proposed methods for detecting CNVs based on comparative genomic hybridization (CGH) [14][15][16][17][18]. In CGH, both a genome of interest (donor genome) and a reference genome are hybridized to a tiling array. The genomes are labeled so that the intensity of each can be differentiated on the array. The ratio of intensities (donor/reference) at each spot provides an estimate of the gain or loss of the genomic sequence represented by the spot. This method, although successful in detecting CNVs, suffers from a number of limitations. The CGH approach lacks the ability to detect CNVs with high resolution. That is, the exact boundaries of the sequence which exists in variable copies are not distinguishable. Also, the CGH approach is unable to detect variations within the genomic copies [4][19]. Nannya et al. [20] and Wang et al. [21] proposed more sensitive methods to improve resolution and genome coverage using whole-genome SNP genotyping arrays. But even these methods lack the ability to effectively reconstruct the regions of interest. In general, array-based methods may theoretically be able to predict the exact boundaries and number of copies of a particular region, but cannot be used to reconstruct the actual region as it appears in the donor genome.

The rising availability of next-generation sequencing (NGS) technologies offers an alternative way to detect CNVs. Next-generation sequencing provides a large number of short reads, as much as 40x coverage for a human individual. These reads can be mapped to a reference genome, in order to identify variations. A few recent studies have proposed methods to detect CNVs using datasets generated by NGS technologies [19][22][23][24]. Simpson et al. [22], using sequence data generated with inbred mouse strains, attempted to predict occurences of CNVs by using a Hidden Markov Model. Their method breaks the genome into a series of windows and determines the copy number state at each window. Adjacent windows that have the same copy number state are combined to determine the full region of the CNV.

Unfortunatly, the boundary resolution for this method is limited by the size of the window, which is typically at least 1 kilo-base. Chiang et al. [19] used a sliding window approach in order to identify genomic regions that are suspected to contain CNVs and to estimate the location of their boundaries. This method is able to predict the boundaries with greater resolution, because it is not limited by the choice of window size. Both of these methods have successfully identified true CNVs. However, their focus has been primarily on predicting the genomic sequence that exist in variable copies.

Medvedev et al. [23] proposed a method to use discordant paired-end reads to identify structural variations. Discordant paired-end reads are reads mapped to the reference sequence in a way indicative of a structural variation. These discordant reads are clustered to provide high confidence for the occurrence of each structural variation. Medvedev et al. [24] proposed an elegant method to detect copy number variations using paired-end reads. Similar to [23], they first cluster discordant paired-end reads to identify CNV boundaries. Next they build a “donor graph”, which represents the genome as a set of sequence blocks connected by a set of edges. The donor sequence can be reconstructed by walking along the edges of the donor graph. A maximal flow algorithm is applied to estimate the most-likely number of copies for each CNV. However, their method aims to solve the general CNV detection problem and is not specific for solving the CNV reconstruction problem.

In this work, we focus on the CNV detection and reconstruction problem for tandemly organized *de novo* CNVs. This type of CNV was shown to make up nearly 89% of all CNVs with size ≥ 10kb found in the mouse genomes [25]. Tandem CNVs have the properties that there are no gaps or very short gaps between the copies and there is only one copy in the reference CNV, while there are multiple copies in the donor sequence. Therefore, according to the definition of Tandem CNVs, we are only trying to detect CNV gains but not CNV losses, since there can be only one copy of CNV in the reference sequence. This structure allows us to efficiently reconstruct the exact CNV copies. We call the CNV in the reference sequence *reference* CNV and the CNV in the *donor* sequence donor CNV. Each copy in the donor sequence can potentially have a different beginning and ending position (prefix and suffix), which can be considered as insertions and deletions. We detect CNVs by examining the mapping structure across the genome using discordant paired-end reads. A discordant read pair is one that maps to the reference in a way that suggests a CNV. These discordant read pairs serve as a signal for potential CNVs. We interrogate the regions in which we observe discordant pairs, in order to determine if a CNV is likely to have occured.

Discordant pairs are clustered to obtain estimates of the CNV boundaries and read coverage is used to estimate the number of copies that exists in the donor. Unlike all the previous methods, our reconstruction algorithm utilizes unmapped reads in order to identify the exact boundaries of each of the predicted copies and subsequently reconstructs the CNV regions as they appear in the donor sequence. For each detected CNV, we estimate both the number of copies as well as the boundaries (breakpoints) of each copy in a donor genome. We then use these estimates in order to generate a reconstruction of the CNV region as it appears in the donor genome.

In order to validate our method, we constructed donor sequences using build 36 of the mouse genome. We chose to use the previous build (current build is 37), because She et al. [25] recently identified a large number of CNV regions within this build. We use this information so that we can avoid known CNV regions and effectively simulate *de novo* CNVs. Avoiding known CNVs, we simulate donor genomes by randomly selecting non-overlapping regions within the reference genome and tandemly inserting multiple copies. We generated a donor genome with 20 chromosomes, each chromosome with 100 randomly inserted CNVs. Our detection method was applied and we found that we are able to accurately identify up to 91% of the inserted CNVs. Also our reconstruction algorithm detects the breakpoints with accuracy up to 97%.

## Results and discussion

### Simulation

In order to test our methods, we developed a simulation framework in which a donor genome is created by altering a given template genome by inserting non-overlapping CNVs. The number of copies for each CNV is selected uniformly at random from between 2 and 4 and the prefix and suffix of each copy is selected uniformly at random within a range that guarantees that the copy will be no smaller than 1000 base pairs.

For each of the resulting donor genomes, paired-end reads are simulated. The reads are of length 36 bp and the inserts are chosen between 90 and 100 bp, uniformly at random. The full set of reads are mapped to the original reference genome using the MAQ mapper [26] and the resulting MAQ mapping files are then used by our detection and reconstruction methods.

We used this framework to generate one full mouse genome with 20 chromosomes, using build 36 of the mouse genome. For each chromosome we inserted 100 non-overlapping CNVs, avoiding regions with known CNVs, as described by [25]. Each generated genome contains 20 chromosomes each with 100 inserted CNVs, resulting in 2,000 CNVs in total. Each donor chromsome resulted in over 100 million reads. Table 1 summarizes the number of CNVs simulated within each length range, as well as the number of these belonging to each copy-count category.

**Table 1:** Summary of simulated CNVs

CNV Length	Number	Copy-counts (2,3,4)
*l* ≥ 1, 000, 000	80	31,26,33
500, 000 ≤ *l* < 1,000, 000	390	120,139,131
100, 000 ≤ *l* < 500, 000	975	319,335,331
50, 000 ≤ *l* < 100, 000	217	70,79,68
10, 000 ≤ *l* < 50, 000	247	78,89,80
5, 000 ≤ *l* < 10, 000	47	15,14,18
1, 000 ≤ *l* < 5,000	44	12,15,17

The number of CNVs belonging to each length class along with the number of copy-counts for each range are given.

### CNV detection

Medvedev et al. [24] illustrate that the Formula 1 works well on Yoruban HapMap individual NA18507. We next show that this formula may not always work. For example, when the CNV copies have very different length, the likelihood function defined in Formula 1 usually does not return the correct copy-counts. We applied our detection algorithm to the set of 20 chromosomes containing 2,000 CNVs as described in section . When considering the fraction of detected CNVs in each chromosome, we find that our method is able to effectively detect on average 88.5% of the inserted CNVs, while accurately predicting the *copy-counts* for 83% of these. Table 2 summarizes the results obtained by our detection algorithm for varying coverage ratios as well as varying copy-counts. We show that as the copy-count increases, our algorithm has decreased power to detect both the presence of a CNV as well as the true copy-counts. Our algorithm has a slight decrease in detection accuracy when the coverage ratio is reduced, but maintains roughly constant power to predict the true copy-counts over varying coverage ratios.

**Table 2:** Summary of the percentage of detected CNVs and predicted copy-counts broken down by true copy-counts

Copy-Counts	Detected			Predicted copy-counts		
	C=40	C=30	C=20	C=40	C=30	C=20

2	91.2%	91.0%	90.7%	89.2%	89.7%	89.7%
3	87.9%	87.5%	86.6%	87.9%	88.6%	88.5%
4	86.7%	85.1%	84.7%	72.7%	71.7%	72.2%

CNVs were considered to be detected when the begining and ending reference CNV positions were predicted within 10 base-pairs. In practice, the average deviation from the true positions was about 2 base-pairs. The percentage of predicted-copy counts is reported as the percentage of detected CNVs for which were able to accurately predict the true copy-counts. The average running times for the detection algorithm are 552s, 527s and 497s for coverage of 40, 30 and 20, respectively.

We found that most of the missing CNVs are in regions containing repeats. And for those which we are not able to predict the copy-counts, we found that the number of reads mapping to the reference CNV region was much smaller than expected given the length of the reference region and the true copy-counts. We examined the ratio of the total length of the CNV region in the donor to *l* × *n* where *l* is the length of the reference CNV, *n* is the copy-counts, and found that for CNVs for which we could not detect the copy-counts this ratio was smaller when compared with the CNVs for which we could predict the copy-counts (mean of 0.82 vs. 0.86). Upon examining the distribution of these ratios, we find that their means are significantly different by t-test (p-value ≈ 10^-15^). As described in the methods section, for each CNV the lengths of the copies were generated randomly. Given this, we can expect that as the number of copy-counts increases, the chance to have at least one copy that is significantly smaller than *l* increases. If this is the case, then we expect that on average our power to predict the true copy-counts will decrease as the number of copy-counts increases. This is what we observe and have summarized in Table 2. Therefore, when the length of the copies varies too much, the Formula 1 has relatively low prediction power for the copy-counts. When the CNV copies have similar length, such as those in Yoruban HapMap individual NA18507, our method can detect both the CNV and the copy-counts with high accuracy.

### CNV reconstruction

Once the predicted CNVs have been obtained, we attempt to reconstruct them as they appear in the donor sequence. We expect that reads spanning the junction between two adjacent copies will not map to the reference sequence. We utilize these reads in order to find the exact boundaries between copies. Given the high coverage of the next generation sequencing data, each junction should be spanned by a at least one read. We can split these unmapped reads and try to map both parts to the reference sequence. If both parts map perfectly to the reference sequence (for simplicity, we assume all the reads are error-free), the mapped position indicates the corresponding start and end positions of the two adjacent copies.

We take the correctly identified CNV regions and their copy-counts from the previous step and then apply the reconstruction method to identify all junctions. To evaluate the accuracy of the reconstruction, we evaluate the accuracy of the identified junctions. We consider an identification as accurate if the identified position is within 100 bps of the true junction position. Given a junction a|b, we do not require that a successful identification always identifies both *a* and *b* correctly. Instead we allow partial identification, namely if we only identifie *a* successfully, we consider the identification of *a* as a success and the identification of *b* as a failure. Therefore for all junctions, we calculate the percentage of the successful identifications. We summarize the performance of our method in Table 3 and 4.

**Table 3:** CNV Length vs. Coverage Ratio vs. CNV junction identification accuracy

CNV Length	Accuracy			Run Time (sec.)		
	C=40	C=30	C=20	C=40	C=30	C=20

l ≥ 1,000,000	72.35%	68.6%	67.8%	968.59	228.39	93.6
500,000 ≤ l < 1,000,000	79.35%	78%	77.5%	189.38	100.25	60.17
100,000 ≤ l < 500,000	84.10%	84.8%	83.2%	3.71	3.42	3.04
50,000 ≤ l < 100,000	82.3%	82.3%	88%	0.01	0.01	0.01
l < 50,000	96.7%	96.7%	96.7%	0.016	0.014	0.014

*l* is the length of the CNV in the reference, *C* is coverage ratio.

As we can see in Table 3, the smaller the length of the CNV region is, the higher the accuracy is. This is because longer regions are more likely to contain longer repeats. Thus even if we split the unmapped reads into two long enough parts, these parts may still randomly map to the repeat region. For CNV of length less than 50,000, the accuracy of our method is very good. We also show the averaged run time of the method. The run time is just the time for junction validation and donor sequence reconstruction. It does not include determining the reads are from which CNV since this process depends on MAQ. As we can see, generally speaking, the longer the CNV is, the more time consuming the method is. However, the run time also depends on the sequences themselves, in that the sequences with more mapping positions require longer time to process. But generally speaking the averaged processing time for the CNVs is very short. In order to show how the coverage ratio affects the reconstruction algorithm, we also simulated data with 30 and 20 times coverage ratio, respectively. The results are shown in Table 3. As we can see, generally speaking, the higher the coverage ratio is, the better the reconstruction accuracy is. And the reconstruction accuracy also depends on the CNV sequence itself. If the CNV sequence contains more repeats, which leads to more mapping positions, the accuracy drops. That’s probably why we observe a performance drop for coverage ratio 40 and 30 when 50, 000 ≤ *l* ≤ 100, 000. As to the run time, the lower the coverage ratio is, the shorter the run time is, since we need to process fewer reads.

In Table 4, we observe that generally the accuracy increases as the copy-counts decreases. However, the accuracy for copy-counts 3 is slightly higher than the accuracy for copy-counts 2. This is because the average length for CNV with copy-counts 3 is much shorter than that for CNV with copy-counts 2. As we showed in Table 3, the smaller the length of the CNV region is, the higher the accuracy is. When the average length of the CNVs is similar, such as CNVs with copy-counts 2 and 4, lower copy-counts makes the accuracy higher. The average run time shows that the more copies the CNV has, the longer time our method takes.

**Table 4:** Copy Counts vs. CNV junction identification accuracy

Copy-Counts	Accuracy	Average Length	Run time (sec.)
2	77.14%	519,571	79.13
3	79.44%	446,300	115.35
4	73.57%	525,717	1754.72

## Conclusions

In this work, we proposed a novel method to first detect copy number variations (CNV) then to reconstruct CNV copies using paired-end reads generated by the next-generation sequencing (NGS) technology. Our method focuses specifically on tandemly organized *de novo* CNVs, where there is only one copy in the reference sequence and there is no gap or very small gap between copies of CNVs in the donor sequence. We show our method achieves high prediction and reconstruction accuracies on the simulated data sets for tandemly organized *de novo* CNVs. Given roughly 89% of CNVs found in the mouse genome with size ≥ 10kb are tandemly organized [25], our method is practical for mouse genome CNV detection and reconstruction.

## Methods

### CNV detection

#### Discordant paired-end reads

Using next-generation sequencing, short reads are generated in pairs, where a short gap appears between the two reads and the distance of this gap is roughly known. The distance is called *insert length.*

**Figure 1:**
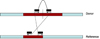
A discordant pair can imply the presence of a CNV.

Discordant read pairs occur when the mapping of a paired-end read is not what is expected, given that there were no structural variations in the donor genome. An example to illustrate discordant read pairs is shown in Figure 1. In this figure, the reference genome contains one copy of the highlighted sequence, while the donor genome contains two tandem copies. A read is sampled from the donor such that the forward read is read from the end of the first copy and the reverse read is read from the beginning of the second copy. When mapped to the reference genome, these reads will map in an unexpected orientation. That is, the reverse read will map upstream of the forward read. By finding reads of this type, we can interogate their regions of mapping to find possible CNVs.

#### Clustering discordant pairs

After mapping a complete set of paired-end reads to a reference genome, we will find many discordant pairs. Given the high coverage, we expect that many discordant pairs will come from each CNV. Therefore, to avoid making duplicate CNV predictions and to bolster our confidence in each predicted CNV, we cluster discordant pairs so that each resulting cluster represents a potential CNV.

We define a simple greedy clustering procedure that clusters discordant pairs that explain the same CNV. We start with the complete set of discordant reads. We select one discordant read *x* from the complete set of discordant reads and find the set {*y*} of all other discordant reads such that the difference in forward read mapping positions for *x* and *y* is no greater than *M_I_*, which is the insert length of the paired-end reads. We call the set of reads *x* + {*y*} *explain* a potential CNV and remove them from the set of all discordant reads. Then we continue this processs until there are either no single discordant reads left in the set of all reads or there are no two single reads such that the difference in their forward read mapping positions is less than or equal to *M_I_*. For each potential CNV, we now have a set of discordant reads and we estimate the boundaries by examining the set of mapping positions of these reads. We estimate the leftmost boundary *b* by taking the minimum of the reverse read mapping positions and the rightmost boundary *b* + *l* by taking the maximum of the forward read mapping positions.

#### Estimating copy-counts

Given a predicted CNV region *c* and the set of reads mapped to this region in the reference, we would like to determine how many copies *d* of the reference region are contained in the donor sequence. In order to determine *d*, we first define a function to calculate the likelihood of observing *r* reads within a region of length *l*, given a particular coverage level. For a region of length *l*, the probability of a read mapping to this region is  where *G* is the length of the genome. We can calculate the probability of *r* reads mapping to the length *l* region by using the binomial distribution. Therefore, the expected number of reads mapped to a length *l* region is  where *N* is the total number of reads generated from the whole genome. When *N* is very large, the binomial distribution can be very well approximated by the Poisson distribution.

Therefore, we can use the density function for the Poisson distribution with  in order to calculate the probability of *r* reads mapping to a length *l* region.

We expect that the length *l* region is represented *d* times in the donor sequence. However, we do not know *d.* We have only observed the number of reads mapped to the length *l* region in the reference. Given this we define the following likelihood function.(1)

By finding the *d* that maximizes the likelihood of *r* reads mapping to the reference region *c*, we determine the number of copies of *c* contained in the donor.

Medvedev et al. [24] applied the same likelihood function defined in the Formula 1 and illustrated that it works well on Yoruban HapMap individual NA18507. Next we show this likelihood function may not always work. If we define *length(donor)* as the total length of the donor CNV, we have  where *d* is the copy-counts, *l*_*i*_ is the length of the *i*-th copy and we define *l* as the length of the reference CNV. The likelihood function assumes all the copies in the donor sequence are of similar length, namely *I*_i_*≈ l* for all 1 ≤ *i* ≤ *d.* Then *length(donor) ≈ d x l.* However, when the length of the copies differs from each other too much, namely the prefix and suffix of some *i*-th copies are big enough such that *l_i_* « l, *length(donor)* may decrease significantly from *d x l.* Then we may have *||((d − k) x l) − length(donor)|| < ||(d x l) − length(donor)||* for some *k* ≥ 1, which implies that there is at least one copy of length less than l. Otherwise *(d x l) − length(donor)* should be 0. If the donor CNV is of length much less than *d x l,* the true copy-counts would not maximize the likelihood function defined above and the copy-counts estimated will be inaccurate. We show later in our experiments that when the assumption that all the copies in the donor sequence are of similar length is violated, the likelihood function fails to identify the true copy-counts.

### CNV reconstruction

Once the CNV region is detected and the CNV copy-counts are estimated, we want to reconstruct the exact CNV copies as they appear in the donor sequence. We call the CNV in the reference sequence *reference CNV* and the CNV in the donor sequence *donor CNV.* The donor CNV can be denoted as [*p*_1_, *s*_1_ ][*p*_2_ ,  *s*_2_ ]...[*p*_*n*_,  *s_n_* ], where *n* is the copy-counts, [*p_i_*,  *s_i_* ] indicates the *i*-th copy, *p_*i*_* indicates the corresponding start position of the *i*-th copy in the reference sequence, *s_i_* indicates the corresponding end position of the *i*-th copy in the reference sequence. We define s*_i_*|*p_i_*_+1_ as the junction between the *i*-th and *i* + 1-th copy. The CNV Reconstruction problem thus can be stated as following: Given copy-counts *n*, reference CNV region [*b*, *b* + *l*], where *b* is the starting position of the reference CNV and *l* is its length, identify all *p_i_* and *s_i_* for 1 ≤ *i* ≤ *n* and order them such that the number of mismatches is minimized when all the reads are mapped to the reconstructed sequence.

**Figure 2:**
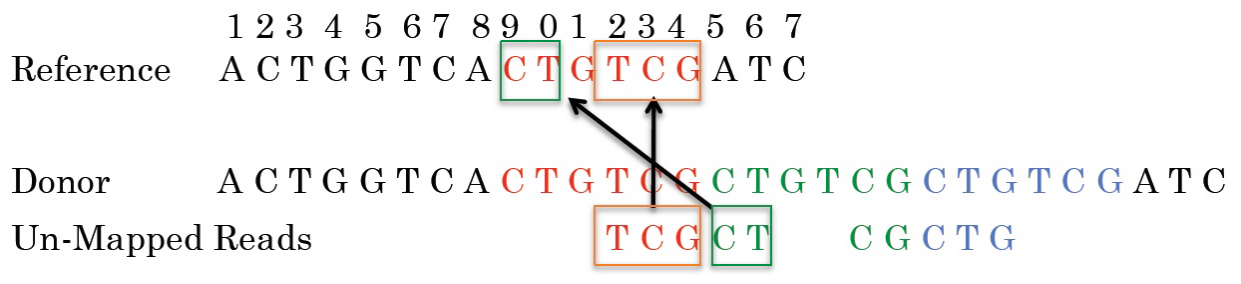
**An example for reconstruction CNV**.The reference CNV is “CTGTCG”. The CNV is copied three times in the donor sequence.

We use unmapped reads to detect the exact junctions of the copies. Given the high coverage ratio of the next generation sequencing data, each junction should be spanned by certain amount of reads. These reads won’t map to the reference sequence and they indicate the start and end positions of the corresponding copies. We can split these unmapped reads and try to map both split parts to the reference sequence. If both parts map perfectly to the reference sequence (for simplicity, we assume all the reads are error-free), the mapped position indicates the corresponding end and start positions of the two adjacent copies. We show a simple example in Figure 2. As we can see, the reference CNV “CTGTCG” is copied three times in the donor sequence. The unmapped read TCGCT doesn’t occur exactly in the reference sequence and it spans the junction between the first and the second CNV copy in the donor sequence. If we split the read into two substrings TCG and CT, both of them map perfectly to the reference CNV. The end mapping position of TCG indicates the first copy ends at position 14 in the reference. The start mapping position of CT indicates the second copy starts at position 9. Next we show the detailed reconstruction process.

#### Unmapped reads Identification

Since we are using unmapped reads to identify the boundaries of CNV copies, we first need to identify unmapped reads sampled from the CNV region. A naive method is to take all the unmapped reads, split them and map them to every identified CNV region. However, the sequencing technique may introduce a large number of unmapped reads due to sequencing errors. It is very inefficient if we take all of these reads and map them to every CNV region. The problem can be alleviated by using mapped reads. Although we don’t know which donor CNV these unmapped reads come from, we can utilize the other parts in their corresponding paired-end reads to validate which donor CNV they are from. We first identify all the reads mapped to the reference CNV region, which can be done efficiently using any reads mapping tool such as MAQ [26]. Next we check the other parts of these reads and select all the unmapped ones, which are highly possible generated from the corresponding donor CNV region. The number of such reads is much smaller than the total number of unmapped reads. We are then able to focus on only the unmapped reads generated from the correct donor CNV region.

#### Junction validation

We split the unmapped reads and map them back to the reference sequence, as shown in Figure 2. A junction is valid only if it is validated by at least one unmapped reads. Therefore we can split an unmapped read at each internal position, which results in two split substrings *r*_1_, *r*_2_. *r*_1_ is the suffix of the first copy and *r*_2_ is the prefix of the second copy. We then map the two substrings back to the reference CNV. If both substrings mapped perfectly to the reference CNV (again, assuming the reads are error-free), the mapping positions in the reference CNV of *r*_1_ and *r*_2_ indicate the corresponding end and start positions of the two adjacent copies.

One problem for the validation is if the length of the split substring is short, it’s highly possible that the split substring maps perfectly to the reference sequence by random chance and the substring may map to many positions. For example, in Figure 2, the unmapped read TCGCT is split to TCG and CT. The substring CT maps to both position 2 and 9. To address the problem, we define a *significant length threshold t* such that when we split the read, we only split the read at positions where the resulting two substrings are of length both no less than *t.* Alternatively speaking, we split the read at the positions in the range of [*t, m-t*] in the read, where *m* is the read length. Therefore a perfect map of a split substring to the reference sequence is highly unlikely occurring by chance. We call such a mapping a *significant mapping.* If a splitting results in two substrings which both have significant mappings, we call such a splitting a *significant splitting.* The significant threshold can be determined by the following formula:(2)

where *L* is the length of the reference CNV region, *∈* is a small number, such as 0.05. The formula indicates that we use the minimum *t* such that the expected number of any length *t* substring occurring in the CNV region is no more than *∈*. For example, in Figure 2, *L*=17. If *∈* = 0.05, according to Formula 2, we obtain *t* ≥ 4. Therefore, the length of the substring as what we used in the example (length as 3 and 2 for the two split substrings) is too small to avoid possible mappings by random chance.

#### Donor sequence reconstruction

Given any order of the junctions, we are able to reconstruct a donor CNV. For example, if the order of the junctions is: *s*_1_ |*p*_2_, *s*_2_|*p*_3_, ..., *s*_*n*-1_ |*p_n_,* the reconstructed donor CNV is [*s, s*_1_ ], [*p*_2_, *s*_2_ ], ..., [*p*_*n*-1_, *S*_*n*-1_ ], [*p_n ,_ e*], where *s* and *e* are the start and end positions of the CNV, respectively. However, not all the donor CNVs are valid. We need to follow a simple rule: for any two adjacent junctions *s_i_*|*p*_*i*+1_ and *s*_*i*+1_ |*p*_*i*+2_ , we need to have *p*_*i*+1_ ≤ *s*_*i*+1_ , namely the starting position of a copy should be no greater than the ending position of the copy. For example, if we only have two adjacent junctions 100|200 and 150|50, the order of the two junctions can be only 150|50, 100|200. The corresponding reconstructed donor CNV is thus [*s*, 150][50, 100][200, *e*], where *s* and *e* are the start and end positions of the CNV, respectively. If we have order as 100|200, 150|50, the reconstructed donor CNV is [*s*, 100][200, 150][50, *e*], where the middle copy [200, 150] is invalid.

Notice there can be multiple orders satisfying this rule and therefore the donor CNV may not be unique. For example, given junctions 100|200 and 300|50, *s* and *e* as the start and end positions of the CNV, we can have an order 100|200 and 300|50, where the reconstructed donor CNV is [*s*, 100][200, 300] [50, *e*], or an order 300|50 and 100|200, where the reconstructed donor CNV is [*s*, 300][50, 100][200, *e*]. Both reconstructed donor CNVs are valid. We are not able to find the true donor CNV given the paired-end reads only. Therefore, we simply output all the possible donor CNVs.

When the CNV region contains repeats, there might be false positive junctions predicted using the above method. We simply rank all the predicted junctions using the number of unmapped reads that can validate them. We pick the junctions with the highest ranks. The number of junctions selected depends on the copy counts.

## Authors contributions

Dan He developed and implemented the algorithm for CNV reconstruction. Nicholas Furlotte developed and implemented the algorithm for CNV detection. The manuscript is a joint work by Dan He, Nicholas Furlotte and Eleazar Eskin.
